# Emirbayer and Desmond's work on The Racial Order: A commentary

**DOI:** 10.3389/fsoc.2023.1010967

**Published:** 2023-03-22

**Authors:** Ayodeji Bayo Ogunrotifa

**Affiliations:** Royal Holloway, University of London, Egham, United Kingdom

**Keywords:** Racial Order, racism, racialisation, capitalism, ruling class

## Introduction

Emirbayer and Desmond's text on The Racial Order (2015) provided an interesting account that seeks to articulate how race is embedded in social relations and constitutes the fabric of institutional and social interactions in everyday life. The text provided an important theoretical tool to map out the structures of racial actions, dynamics of racial field, the dimensions of racial conflicts and consensus that pervaded the landscape of American social life, using Deweyan Pragmatism, Durkheimian sociology and Bourdieusian philosophy to theorize around reflexivity, relationality, and reconstruction. However, the scrutiny of The Racial Order, seven years after its publication, had revealed holes at the center of theorizing; and those holes consist of the limited understanding of the contemporary expression of racism and the problematic nature of the programme of action—color blindness, multiculturalism and racial democracy—that seems more relevant at the individual level, but remains ineffective and illusory in relation to the structural context in which racism is experience, practiced and reproduced. These two issues are what this paper seeks to articulate below.

First, The Racial Order does not capture the broader pattern in which racism is experienced and practiced in the contemporary global context. Emirbayer and Desmond defined The Racial Order as “an organized social relations, symbolic classifications, and even collective emotions” that came into existence in terms of a white/non-white polarity (Emirbayer and Desmond, 2015, p. 15). Therefore, The Racial Order is a framework of a “complex of social-structural, cultural, and collective-emotional matrices of relations” (Emirbayer and Desmond, 2015, p. 83) of racial groups. These racial groups are “races” that occupied a racial field and possessed racial capital. These racial fields are “organized in terms of the structure of distribution of different types of capital or assets, the most important being specifically racial capital” (Emirbayer and Desmond, 2015, p. 88). The racial fields, as Emirbayer and Desmond later continue, are “two poles that are of racial dominance, occupied by racial groups with asset structures featuring a high volume of economic, cultural, and especially racial privilege, and that of racial subordinance, occupied by dominated racial groups with asset structures having a lesser quantity of the above” (Emirbayer and Desmond, 2015, p. 88). If Emirbayer and Desmond's description of The Racial Order is to be restated in another form, racism could be defined as matrices of relations involving two “races”—those who have a racially dominance and those who are racially dominated. The key issue here is that the current nature of racism transcends beyond “race” (biological categorization) that shaped the articulation of racial dominance and racial subordinance that featured prominently in The Racial Order.

Racism, as articulated in The Racial Order, could be said to have derived from Banton's (1970) articulation of racism as the doctrine of racial typology. The definition of racism, from Banton's perspective, as ‘doctrine that a man's behavior is determined by stable inherited characters deriving from separate racial stocks having distinctive attributes and usually considered to stand to one another in relations to superiority and inferiority' (Banton, 1970: 18), reduced racism to a biological categorization and obscured the question of who utilizes racism as a doctrine or whose doctrine was racism. Banton's definition has been challenged by Robert Miles, whose corpus of work (Miles, 1982, 1989, 1993) argued that racism is an ideology of racial categorization. Miles argued that racism is an ideology that cannot be reduced to a biological categorization.

Using the example of anti-Irish racism in the UK, Miles argued that racism is not solely based on biological classification, but also includes religious superiority. Subsequent studies by Mellinkoff (1993), Stoler (1997), Strickland (2003), and Whitaker (2015) have demonstrated that racism is not based on biological categorization, but cultural classification. Using the examples of racism against gypsies and travelers and anti-moor (Muslim) racism in the feudal Europe, these studies have confirmed Miles' position that racism is more than biological (racial) categorization. However, Mike Cole in his corpus of studies (Cole, 2009a,b, 2016, 2020) expanded the analyses of racism to accommodate the contemporary issues of xeno-racism (non-color coded racism) or newer hybridist racism such as Islamophobia, anti-asylum-seeker racism, anti-refugee racism, anti-Asian racism (especially Chinese), and racism against Polish and other Eastern Europeans in the UK. Cole's works have revealed how the territorial landscape of racism has shifted from race (biology), culture and religion to racism that is now based on nationality (anti-Polish, anti-Chinese, and anti-Eastern Europeans in the UK), language, and immigration status (against refugees and asylum-seekers). The implication of Cole's work is that what constitutes racism now is not only by referring to superiority or inferiority based on “race” (biology), culture and religion, but also includes making reference to superiority or inferiority based on the nationality, language, and immigration status of individuals or groups. These scholarly positions have demonstrated a broader understanding of racism beyond the notion of race and demonstrates that The Racial Order does not have the purchase of the current understanding of racism and what constitutes racist actions in the contemporary global context.

The second issue is the programme of action articulated in The Racial Order. Emirbayer and Desmond proposed color blindness, multiculturalism, and racial democracy as the programme of action to resolve the problem posed by racism, especially in American society. These concepts represent the forms of racial reconstruction needed to achieve stability of The Racial Order. This proposal could only be achieved at the individual level of experience and only limited to individuals but does not capture the larger and structural context in which racism is practiced and reproduced in Western societies (American society inclusive), which is racialisation. Racialisation has been defined “as an ideological process that involves racialising benefits, privileges, and opportunities to one group [possibly an ethnic group] over other groups by the capitalist ruling class and the state, and legitimizing it by using policies, media, laws, regulations, and institutional practices as a means of entrenching division and disunity in the society and preserving their system of control under capitalism” (Ogunrotifa, 2022, p. 240). This definition of racialisation has demonstrated that overt references to inferiority, superiority, distinct races, and racial hierarchy are rare in the contemporary era, especially in the public space, as racism is still practiced and reproduced covertly through racialisation. Although racist tropes and dog whistles rhetoric are often deployed against immigrants, refugees and asylum-seekers by politicians, far-right groups, right-wing organizations, and others (Cole, 2016), and such a form of racism is often downplayed or rationalized as a way of protecting the national interests.

With racialisation, racism is usually practiced and reproduced by the ruling class (in this case, the American ruling class) to maintain capitalist control. The process of racialisation occurs when the ruling class activates racist ideology to legitimate exclusionary practices to protect their class rule and the capitalist system, using policies, media, laws, regulations, and institutional practices. The modern expressivity of racism and exclusionary practices are covert, hidden, and disguised, as the mechanism of exclusion and racialisation are concealed in any policy, law and regulation. This was even confirmed by Emirbayer and Desmond in that racism manifests in “state policies, economic disparities, discrimination in the legal system, and institutional racism in formal settings of all conceivable kinds” (Emirbayer and Desmond, 2015, p. 232).

The process of racialisation, as Miles (1989, 1993) and Webster (1993) observed, is being practiced through allocative mechanisms. Through allocative mechanisms, there might be low budgetary allocation to poor areas inhabited by Blacks and other ethnic minorities, as opposed to rich areas inhabited by the White and super-rich people, by the ruling class and its allies in governmental institutions. The distribution of opportunities and the different types of capital or assets that Emirbayer and Desmond (2015, p. 88) noted is the distribution of the benefits of capitalism across racial groups by the American ruling class, in which racialisation is central to the distribution process. With racialisation, a vast gap or considerable differences between the Whites and the minority ethnic groups are observed in a whole range of areas such as housing, employment, occupation, job opportunities, career progression/promotion, education, health, and social deprivation (Cohen, 2001; Li and Heath, 2008; Li, 2017). Furthermore, racialisation has further helped to perpetuate persistent racial inequalities in the rejection of policies meant to alleviate racial inequalities, health and wealth, and criminal sentencing, and the persistent racial discrimination in hiring, credit markets, and housing (Bobo and Hutchings, 1996; Pager and Shepherd, 2008; Fiske, 2010). Racism is therefore being reproduced through racialisation, where barriers to Blacks and other ethnic groups in the United States are artificially created by the American ruling class to maintain capitalist social control (Cox, 1970).

Racialisation is a weapon used by the capitalist ruling class and its representatives in government and other institutions to deepen racism and maintain its hold on the society by racialising one ethnic group against another to prevent class unity, using the media (owned and controlled by the ruling class to racialise and divert peoples' attention from the main problem), state policies, laws and regulations, and institutional practices. The ruling class employs racialisation in policies to pacify the majority White population and gives the latter the impression that they are actually “better” than the Blacks and other minorities, and that there is no alternative to capitalism that has been beneficial to their lives (Collins, 1987; Cohen, 2001; Bakan, 2008; Virdee, 2010). In this regard, racialisation is the method used by the American ruling class, initiated through state and social institutions, to foster division and disunity in society, especially among workers of different ethnicities and to prevent the unity of the working people along the class line, which is seen as inimical to the capitalist system of control.

In returning to the reconstruction proposal of color blindness, multiculturalism, and racial democracy, Emirbayer and Desmond's programme of action is the quest to resolve the problems posed by racism, or the “racial problem,” within the framework of capitalism. The proposal seems illusory if capitalism and role of the ruling class (vis-à-vis slavery and colonialism), which are the root cause of racism in American society, is not addressed. The proposal around color blindness would be ineffective because racist practices in the contemporary context are not overt, in which state institutions, budgetary allocations, appointments, legislations, policies and regulations are covertly racialised. Multiculturalism and racial democracy will be ineffective in addressing racism where capitalism exists. This is particularly salient in American context where the reproduction of racism occurs because of the failure of the capitalist system to provide full employment, free healthcare services, good and affordable housing for all, and a free and world-class education, despite being the richest country in the world in modern history.

If the resources of American society are equally distributed and allocated, there would not be racial and ethnic tension and conflict. This racial and ethnic tension is the fallout of the class question that is rooted in the existence and reproduction of racism beyond the capitalist American society. Under capitalism, the resources of the society are not evenly distributed throughout the society, and this produces inequality among classes (Amin, 2014). This inequality facilitates the reproduction of racism, because the little provided by capitalism to other classes (excluding the ruling class) is being distributed to a section of the population through racialisation. Therefore, in distributing these limited resources and opportunities, racism is activated through the process of racialisation, where exclusionary practices are legitimated in the form of a discrimination against Black and other ethnic minorities. Without racialisation, racism cannot be understood on the terms in which it was articulated in The Racial Order.

Behind the façade of multiculturalism and racial democracy lies not the ethnic question, but the class issue and class control which legitimated the existence and reproduction of racism. Multiculturalism and racial democracy under a capitalist society would be under severe social strain, especially during an economic crisis when the class struggle intensifies. As capitalism always produces class tension (Wright, 2000; Harvey, 2014), this tension will find its expression in conflicts among ethnic groups, jostling for limited opportunities and scarce resources. Therefore, color blindness, multiculturalism, and racial democracy, as Emirbayer and Desmond proposed, cannot resolve the problem of racism in any capitalist society.

## Conclusion

This paper has critically reviewed Emirbayer and Desmond's work on The Racial Order and outlined two gray areas associated with the framework. While racialisation was not discussed in the Emirbayer and Desmond's framework and was not even mentioned in the book. This lacuna thus weakens not only the theoretical utility of The Racial Order in understanding the experience of racism in the contemporary era, but also develops a comprehensive proposal to address the problem posed by racism in all spheres of social life. In conclusion, my argument is that racism is sustained and reproduced through racialisation, and the notions of color blindness, multiculturalism, and racial democracy, as proposed by Emirbayer and Desmond, cannot address the problem posed by racism unless the structural context of capitalism and the role of ruling class, which sustained racism, is challenged. I welcome Emirbayer and Desmond's thoughts and reflections on these matters in the interests of intellectual and constructive exchange that defines the best traditions of academic life. In doing so, we move to develop theoretical and practical programmes to end racism in social life.

## Author contributions

The author confirms being the sole contributor of this work and has approved it for publication.
